# Translation of HIV/AIDS knowledge into behavior change among secondary school adolescents in Uganda: A review

**DOI:** 10.1097/MD.0000000000036599

**Published:** 2023-12-08

**Authors:** Emmanuel Ifeanyi Obeagu, Getrude Uzoma Obeagu, Moses Onyemaechi Ede, Edward Odogbu Odo, Hauwa Ali Buhari

**Affiliations:** a Department of Medical Laboratory Science, Kampala International University, Uganda; b School of Nursing Science, Kampala International University, Uganda; c Department of Education Foundations, University of the Free State, Bloemfontein, South Africa; d School of General Studies (Physical and Health Education Unit) Michael Okpara University of Agriculture, Umudike, Nigeria; e Department of Haematology, School of Medical Laboratory Sciences, Usmanu Danfodiyo University, Sokoto, Nigeria.

**Keywords:** adolescents, AIDS, behavior change, HIV, Knowledge

## Abstract

The human immunodeficiency virus/acquired immunodeficiency syndrome (HIV/AIDS) pandemic is primarily affecting young people worldwide, with those between the ages of 15 and 24 accounting for nearly half of all new infections. This paper was written to effectively translate HIV/AIDS knowledge into actionable behavioral changes among secondary school students in Uganda by empowering them with comprehensive information, fostering a deeper understanding of preventive measures, and facilitating the development of responsible and informed decision-making skills, thereby reducing the incidence of HIV/AIDS transmission within this demographic. There is a relationship between risk perception and behavior change in HIV/AIDS prevention among high school students. This can be explained by the high proportion of secondary school students who think they are at risk of HIV infection; this perception may be related to having had early sex, being sexually active, and knowing someone has died of HIV. High school students regularly engage in risky sexual behaviors, such as not using condoms and having multiple lifelong partners. Student behavior is significantly influenced by HIV and AIDS prevention initiatives such as youth-friendly services, peer education, and condom use.

## 1. Introduction

Human immunodeficiency virus (HIV) is a viral infection that is mainly spread through sex, sharing objects with sharp edges, such as razor blades, or from an infected mother to her unborn child. HIV infection impairs the immune system of the body, making it more susceptible to many different infections as well as the emergence of specific cancers.^[1–3]^

When HIV symptoms are severe, the illness is called acquired immunodeficiency syndrome (AIDS) because the virus remains in the body permanently once it has been contracted.^[4]^

AIDS is caused by the serious HIV pandemic that the world is currently experiencing.^[5]^ It is one of the most dangerous diseases in the world and has claimed the lives of both young and old people due to the lack of a treatment at this time.^[6]^

By providing learning opportunities for everyone to acquire the knowledge, abilities, competencies, values, and attitudes that will limit the spread and effects of the pandemic, including through access to care, counseling, and education for treatment, we are referring to HIV/AIDS education.^[7]^

The HIV/AIDS pandemic is primarily affecting young people worldwide, with those between the ages of 15 and 24 accounting for nearly half of all new infections.^[8]^

The number of people living with HIV worldwide as of 2021 was 38.4 million, of which 36.7 million were adults and 1.7 million were children under the age of 15. A total of 1.5 million people (2021), including 1 point 3 million adults and 160,000 children under the age of 15, were newly infected. A total of 650,000 HIV/AIDS-related deaths were reported in 2021, of which 560,000 involved adults and 98,000 involved children under the age of 15. A total of 2500 new infections occur every day, with 500 affecting people under the age of 15 and 4400 affecting people over the age of 15, with sub-Saharan Africa accounting for 54% of all infections. Out of 4400 new infections in adults, nearly 49% are in women, 32% are in people between the ages of 15 and 24, and 20% are in young women.^[9]^ This paper was written to effectively translate HIV/AIDS knowledge into actionable behavioral changes among secondary school students in Uganda by empowering them with comprehensive information, fostering a deeper understanding of preventive measures, and facilitating the development of responsible and informed decision-making skills, thereby reducing the incidence of HIV/AIDS transmission within this demographic.

## 2. Methodology

The literature search for “From Awareness to Action: Translating HIV/AIDS Knowledge for Secondary Students in Uganda” involved a comprehensive exploration of various academic databases, research repositories, scholarly articles, governmental reports, non-governmental organizations (NGO) publications, and other reputable sources. The search strategy encompassed the following key elements:

1. Database exploration: Utilizing academic databases such as PubMed, ScienceDirect, JSTOR, and Google Scholar to gather peer-reviewed articles, studies, and reports related to HIV/AIDS education, behavioral interventions, and adolescent-focused programs in Uganda.
2. Government reports and policies: Reviewing official documents, national health reports, and policies issued by Ugandan health departments and education ministries concerning HIV/AIDS awareness and prevention strategies tailored for secondary school students.
3. NGO and organizational publications: Exploring publications from NGOs and international health organizations operating in Uganda, focusing on initiatives, programs, and interventions targeting adolescent HIV/AIDS education and behavioral change.
4. Academic journals and publications: Identifying and analyzing relevant academic journals and publications specific to HIV/AIDS education, behavior change theories, and successful interventions within the Ugandan context.
5. Reviewing intervention studies: Analyzing previous intervention studies and evaluations conducted in Uganda or similar socio-cultural contexts, specifically focusing on strategies that effectively translated HIV/AIDS knowledge into behavioral change among secondary school students.
6. Local and cultural context: Paying attention to studies and literature that consider the cultural nuances, social dynamics, and local perceptions regarding HIV/AIDS within the Ugandan context, ensuring that educational approaches align with cultural norms and sensitivities.
7. Periodical updates: Ensuring the incorporation of the latest available research and literature up to the date of the literature search to provide a comprehensive understanding of current practices, gaps, and emerging trends in HIV/AIDS education for Ugandan secondary school students.

The search strategy aimed to gather a diverse range of scholarly articles, program evaluations, empirical studies, and reports to inform the development of an effective program that translates HIV/AIDS knowledge into actionable behavioral changes for secondary school students in Uganda.

### 2.1. HIV/AIDS perceived risk

The possibility of a negative outcome for a person health, their quality of life, or the environment is known as risk. Risk is defined by the World Health Organization as either a factor that increases the likelihood of a negative outcome or a probability of such an outcome.^[10]^

Risk perceptions are multifaceted and intricate, and they may include judgments about the gravity or seriousness of HIV infection, the risk controllability, or the likelihood that the risk will be experienced.^[11]^

A key factor in explaining why HIV prevention interventions is not used is risk perception, which suggests that when relationships and situations are viewed as low-risk, people are less likely to use and adhere to prevention measures. For epidemiologists, researchers, and healthcare providers, the fundamental idea of risk is the likelihood that an event will occur. People are, however, also at risk for the negative side effects of interventions, such as the physical, social, and psychological risks connected with their use. These could be severe repercussions like stigma or intimate ones like ending a relationship.^[12]^

Concerns have been voiced about risk compensation, or the rise in risky behaviors caused by a decrease in either real or perceived disease risk, throughout the development of HIV prevention interventions, from condoms, pre and post exposure prophylaxes, voluntary medical male circumcision, to emerging interventions, including microbicides, vaginal rings, and injectable antiretrovirals.^[12]^

As a key indicator of HIV risk behaviors, perceived risk has drawn a lot of attention. Perceived risk is a crucial construct in understanding why people engage in behaviors that increase their risk of contracting HIV or how to change these behaviors, according to models like the Protection Motivation Theory, Health Belief Model, Social Cognitive Theory, Extended Parallel Process Model, and AIDS Risk Reduction Model.^[13]^

According to Osingada et al,^[14]^ a large percentage of secondary school students in the Wakiso district believed they had an increased risk of contracting HIV. This perception was linked to early sexual activity, knowing someone who had died from the virus, and knowing someone who had died from it. Risky sexual behaviors are common among secondary school students in Wakiso Town Council, including not using condoms, having multiple lifetime partners, and having sex after consuming alcohol. Therefore, there is a need to develop and implement focused school-based HIV risk reduction programs, according to reports. The findings mentioned above suggest that Wakiso Town Council current school-based HIV prevention interventions may have gaps.^[15]^

As relationships develop and trust is felt, risk perception declines. According to reports, condoms are more acceptable in casual relationships without commitment or fidelity expectations. It became harder to maintain continued use as relationships grew more serious.^[16]^

49.6% of the participants believed they were at risk of getting HIV. The perceived risk of contracting HIV was negatively correlated with being female and not having taken an HIV test. Having ever had sex, being older than 17, knowing someone who died of HIV, having had sex young, and knowing someone who died of HIV were all, however, positively correlated with perceived risk of getting HIV. The only remaining statistically significant event was knowing someone who had died from HIV.^[14]^

Continued use was challenging to maintain as relationships grew more committed. It was discovered that the trust between sexual partners, developed over time, persuaded people to put the relationship and their emotional wellbeing ahead of HIV prevention.^[12]^

Loffredo and Opt (2015) discovered that gender and ethnicity are key factors in how vulnerable college students perceive their risk of contracting HIV. According to Loffredo and Opt research, men tend to perceive risk differently than women do. The 2017 National Survey of Young Adults conducted by the Kaiser Family Foundation and these results are in agreement. Loffredo and Opt also found that having close personal acquaintances who were HIV-positive or had passed away from AIDS increased one perception of vulnerability to HIV infection. Even when they engage in risky sexual behaviors, all young people generally seem to perceive their risk of contracting HIV as low.^[17]^

### 2.2. HIV/AIDS education and behavior prevention programs in schools

HIV/AIDS education is the process of educating people about HIV/AIDS, including the disease transmission mechanisms and infection prevention strategies.^[18]^ Additionally, it entails empowering individuals with the know-how to apply and practically act upon this knowledge. The vast majority of young people who are at risk attend school. The school system also brings together parents, the community, teachers, and students. A larger audience is thus reached if AIDS information and sex education are taught in schools. Furthermore, it is asserted that education empowers and equips people, particularly young women, to comprehend and internalize pertinent knowledge and to transform knowledge into behavioral change.^[19]^

According to the ABC approach, the main goal of HIV/AIDS education in Uganda is to change people behaviors for the better through intensive information, education, and communication. One of the guiding principles of Uganda sector policy on HIV/AIDS prevention, which was drafted in 2004, was to mainstream HIV/AIDS into all sector-specific policies, procedures, practices, and programs. The sector response also entails the creation of a sector policy that directs all HIV interventions, workplace interventions, prevention, education through schools and institutions, capacity building, the distribution of facts based on evidence about the effects of HIV/AIDS on the sector, and finally the management of the response through effective coordination mechanisms within the education sector at the national and district levels.^[20]^

HIV/AIDS education is also being carried out in Uganda by a number of NGOs. The Family Planning Association of Uganda, which also conducts sensitization campaigns in schools, and the Uganda Program for Human and Holistic Development, which is involved in training pre-service and in-service teachers in the implementation of PIASCY in some districts of the country, were among the first NGOs to get involved.

The primary goal of Straight Talk Foundation was to protect youth from AIDS through communication.

In the effort to combat the epidemic among young people in the nation, other international NGOs like Action Aid and Save the Children have collaborated with other groups and line ministries.^[21]^

In 2009, a 3-day training for 2 teachers, including the head teachers, was held by the Presidential Initiative on AIDS Strategy Communication to Youth, the organization in charge of training teachers. After each lesson, the trained teachers were expected to impart their knowledge and skills to other teachers and students as Trainers of Trainers.^[22]^

There were 5 schools where Straight Talk, a group that promotes safer sex practices like condom use, non-penetrative sex, and abstinence, was present. Five of the schools reportedly have peer-to-peer programs that prepare students to serve as peer educators. Three schools benefited from interventions made by the Kabalore Youth Alliance, a program that educates youth in the Kabalore district about HIV/AIDS and conducts HIV testing among them. The Teen Star program, which mainly teaches about sexuality, helped one of the schools. For a year, the program targets third-year students who are between the ages of 15 and 17. The senior female teacher was given training and a syllabus to help with program implementation. Students meet once a week for the duration of the program first year to discuss a specific topic until the syllabus is finished.^[22]^

Information, education, and communication resources on HIV prevention issues were distributed to 7 schools in Kabalore, along with questions and answers about the experiences of teenagers from across the nation. The use of MDD clubs as information sources in schools is also mentioned.^[22]^

## 3. HIV/AIDS knowledge and behavior change

### 3.1. HIV/AIDS knowledge

A significant obstacle to the eradication of this scourge is the lack of knowledge about HIV/AIDS among young people around the world. Adolescence is when most people start having sexual relations.

In the fight against HIV/AIDS, it crucial to acknowledge that young people are engaging in sexual activity but lack the knowledge necessary to protect themselves.^[23]^

According to study results conducted in secondary schools in central Uganda, most participants knew about the ABC HIV prevention strategy, with rural schools knowing more about it (47%) than urban schools (36%).^[24]^

The results of the study conducted in secondary schools in Buikwe and Kampala revealed that, on average, participants had a 95.1% excellent awareness of HIV/AIDS compared to the 4.9% who were not knowledgeable. In comparison to urban schools, which had a lower percentage of participants who were knowledgeable about HIV/AIDS (46.1%), rural schools had a higher percentage (49.0%).^[24]^

Different responses were given to the modes of HIV transmission by participants in the same study, who attended both rural and urban schools. Sexual activity with an infected person was the mode that received the most mentions (32%), followed by sharing sharp objects with an infected person (4%), oral sex or wound contact with an infected person (2%), and blood transfusion from infected blood (6%).^[24]^

The results also show that the majority of participants in both urban and rural schools learned about HIV/AIDS and primarily discussed it with their friends 37.6%. But the participants were also able to learn about it and communicate about it from their parents, the media, their teachers, and their siblings. The majority of participants—62.0%—reported that there is no cure for HIV/AIDS; 13.1% claimed there is a cure; and 24.9% said they were unsure of the answer. 41.6% of the study participants expressed the opinion that using condoms lessens the pleasure of sexual activity. The use of condoms, according to 343% of participants, is a sign of mistrust.^[24]^

## 4. Behavior change

Lack of knowledge about HIV/AIDS among teenagers is a significant factor in risky sexual behavior. Although a lot of research is done on how much youth know about HIV/AIDS, there are many inconsistencies in the literature.^[25]^

For instance, according to some experts, a person propensity for safe sexual behavior increases with their level of knowledge about HIV/AIDS.^[26–28]^

Particularly, Lawrence in 1993 study of low-income African American adolescents, it was discovered that few of them knew the truth about HIV testing and condom use. Given the important role that knowledge of HIV/AIDS plays in influencing sexual health and behavior, they recognize that this lack of knowledge is problematic. According to this study, adolescents who are more knowledgeable about HIV/AIDS engage in safer sexual behavior.^[29]^

According to data from a baseline survey on enhancing HIV prevention and psychosocial care and support in secondary schools conducted by Baylor Uganda in May 2011,^[30]^ adolescents engage in unsafe sex at school.

Every beginning and end of the term, 3 to 5 girls who were found to be pregnant were reportedly dismissed from 5 of the 8 schools. Youth engaging in sexual activity because they are curious and want to have sex, adolescent raging hormones, peer pressure, limited or absent parental guidance, parental and sibling examples, mass media, and a lack of money and employment opportunities to support their desired expensive lifestyles were some of the explanations offered for this behavior.^[31]^

As was just mentioned, dissonance is a common theme in literature today. It is critical to further investigate adolescent HIV/AIDS knowledge because there are ongoing debates about the extent of adolescents’ knowledge of HIV/AIDS and how this knowledge influences their sexual behavior. Predictors of said knowledge must be taken into account in order to comprehend the implications of said knowledge.^[32]^

## 5. Conclusion

There is a link between perceived risk and behavior modification for HIV/AIDS prevention among secondary school students. This can be explained by the high percentage of secondary school students who believe they are at risk of getting HIV; this perception is probably linked to having an early sexual debut, being sexually active, and knowing someone who died from HIV. Secondary school students frequently engage in risky sexual behaviors like not using condoms and having multiple partners throughout their lifetimes. Students’ behavior is significantly affected by HIV and AIDS prevention initiatives like youth-friendly services, peer education, and condom use. Due to the lack of effective student interventions and the prevalence of peer pressure and social interactions in schools, preventing HIV infection among students in those settings remains challenging. In order to support school interventions, efforts must center on integrating HIV prevention into the curricula, collaborating with peer educators, as well as with education service providers who spend a significant amount of time with the students while at school.

## Author contributions

**Conceptualization:** Emmanuel Ifeanyi Obeagu.

**Funding acquisition:** Emmanuel Ifeanyi Obeagu.

**Methodology:** Getrude Uzoma Obeagu, Moses Onyemaechi Ede, Edward Odogbu Odo, Hauwa Ali Buhari.

**Resources:** Emmanuel Ifeanyi Obeagu.

**Supervision:** Emmanuel Ifeanyi Obeagu.

**Visualization:** Emmanuel Ifeanyi Obeagu, Getrude Uzoma Obeagu.

**Writing – original draft:** Emmanuel Ifeanyi Obeagu, Getrude Uzoma Obeagu, Moses Onyemaechi Ede, Edward Odogbu Odo, Hauwa Ali Buhari.

**Writing – review & editing:** Emmanuel Ifeanyi Obeagu, Getrude Uzoma Obeagu, Moses Onyemaechi Ede, Edward Odogbu Odo, Hauwa Ali Buhari.
